# Investigation of the lower block rows in the King’s Chamber of the Great Pyramid using ultrasonic testing with shear wave arrays

**DOI:** 10.1038/s41598-026-54151-6

**Published:** 2026-06-18

**Authors:** Benedikt Maier, Amr G. Hamza, Alejandro Ramirez-Pinero, Khaled Taie, Thomas Schumacher, Olga Popovych, Mohamed Elkarmoty, Mehdi Tayoubi, Hany Helal, Christian U. Grosse

**Affiliations:** 1 Department of Mining, Petroleum, and Metallurgical Engineering, Faculty of Engineering, Cairo University, Gamaa Street 1, Giza, 12613 Egypt; 2 Chair of Non-Destructive Testing, TUM School of Engineering and Design, Technical University of Munich, Lichtenbergstr. 2, Garching, 85748 Germany; 3 Rock Engineering Laboratory, Faculty of Engineering, Cairo University, Gamaa Street 1, Giza, 12613 Egypt; 4 UNESCO Chair On Science and Technology for Cultural Heritage, Faculty of Engineering, Cairo University, Gamaa Street 1, Giza, 12613 Egypt; 5 Civil and Environmental Engineering, Portland State University, 1930 SW 4Th Avenue, Portland, 97201 OR USA; 6 Dassault Systèmes, 10 Rue Marcel Dassault, 78140 Vélizy-Villacoublay, France; 7 Heritage Innovation Preservation Institute (HIP Institute), 50 Rue de Rome, 75008 ParisÎle-de-France, France

**Keywords:** ScanPyramids (SP), Great Pyramid of Giza, Non-destructive testing (NDT), Ultrasonic testing (UST), Shear wave echo array, Synthetic Aperture Focusing Technique (SAFT), King’s Chamber (KC), Engineering, Physics

## Abstract

This study investigates the internal geometry of the granite block rows in the King’s Chamber (KC) of the Great Pyramid of Giza using Non-destructive testing (NDT) with ultrasonic testing (UST) using shear wave echo arrays. As part of the ScanPyramids (SP) project measurement campaigns in 2022, the lower two block rows of all four walls were examined using ultrasonic shear-wave echo arrays (PD8000, Screening Eagle Technologies). The measurement strategy initially included rapid overview acquisitions using an 8-channel UST device to identify potential areas of interest. High-resolution, detailed measurements were then performed at these locations, particularly along the north wall (NW) and in the area of block labeled NW9, using a 16-channel configuration built from two UST devices. The data acquired during these campaigns were processed using a Fourier Transform Synthetic Aperture Focusing Technique (FT-SAFT) algorithm based on the general Synthetic Aperture Focusing Technique (SAFT), creating two-dimensional (2D) cross-sectional images that reveal internal reflectors. A reliable determination of reflector depth requires a precise measurement of the shear wave velocity, which was determined from measurements on the surfaces of 45 accessible granite blocks of the two lower block rows of the KC. The whole study was performed on these 45 blocks. The chamber-wide mean value of 3035 m/s ± 90 m/s results in a remaining depth uncertainty of only a few centimeters, arising from variations in stone properties and measurement uncertainties (up to ± 70 mm even for deep reflectors). Images reconstructed using an FT-SAFT enabled the determination of block thickness (i.e., the locations of backwalls), block joints, and the identification of additional internal reflectors at depths of up to approximately 3 m. The 16-channel UST device exhibited an improved signal-to-noise ratio (SNR) and greater penetration depth than the 8-channel UST device, enabling hyperbolic reflectors and anomalies behind individual blocks to be visualized more distinctly. From the reconstructed 2D images of the measurement profiles, a three-dimensional model of the lower two block rows was derived, providing for the first time a systematic view of the internal structure of the granite lining of the two lower block rows of the KC’s walls. The results illustrate the potential of UST in combination with FT-SAFT for the NDT of massive historic stone structures, establishing a basis for subsequent multi-modal NDT analyses and structural evaluations of the Great Pyramid.

**Supplementary Information:**

The online version contains supplementary material available at 10.1038/s41598-026-54151-6.

## Introduction

The Great Pyramid of Giza is among the most extensively studied man-made structures in the world, yet key aspects of its internal structure remain insufficiently understood. This is especially true for the so-called King’s Chamber (KC) inside the Great Pyramid of Giza, whose walls, floor, and ceiling are composed of massive blocks made of Aswan granite^1^. While numerous studies have examined the external geometry^2^, the construction phases^1^^[,3^, and the presence of large internal cavities^4^ behind the faces of the whole Great Pyramid of Giza, the internal structure and thickness of the individual granite blocks and walls of the KC remain largely speculative. Invasive investigation methods, such as core drilling, are not permitted for conservation reasons, leaving NDT techniques, such as UST and Ground Penetrating Radar (GPR), as viable options for obtaining additional information about a large stony structure like the Great Pyramid of Giza, especially the KC itself.

In recent years, various geophysical and NDT techniques have been employed within the SP Project^5^, including muon tomography^6^, GPR and UST investigations^7^, and investigations into the sustainability problems of the entire Giza plateau^8^. Earlier research has focused primarily on large-scale questions, such as the detection of previously unknown cavities, such as the so-called “Big Void”^4^ and the Scan Pyramids North Face Corridor (SP-NFC)^7^^[,9^. In contrast, almost no quantitative data have been available on block thickness, joint geometries, or potential internal discontinuities within the granite lining of the KC. Yet, these parameters are essential for refining structural models of the chamber, assessing stress distributions, identifying potential vulnerabilities relevant to long-term preservation, and enhancing knowledge of the KC’s construction.

UST with shear wave echo arrays^10^ has proven to be an effective method in civil engineering for determining structural thicknesses, detecting voids and cracks, and imaging internal reflectors^11^^[,12^. With the introduction of UST devices and SAFT-based reconstruction techniques^13^, it is now possible to generate high-resolution images of a structure’s interior. In particular, FT-SAFT reconstruction (which coherently evaluates all transmitter–receiver combinations) enables focused imaging of reflectors while suppressing undesired side-wall reflections. Within the SP project, this method has already been successfully used to detect the so-called SP-NFC^7^^9^ and two former unknown anomalies at the Menkaure Pyramids’ eastern face^14^.

Applying such UST measurements to the massive granite blocks of the KC, however, presents specific challenges. In addition to the limited accessibility of the surfaces, the density of the Aswan granite, the surface roughness, and the unique microclimate (high humidity and salt deposits) of the KC (located within a heavily visited monument) also play a role in performing UST. The high humidity and salt deposits cause problems with the acoustic coupling, which is crucial for achieving sufficient shear wave penetration depth to image backwalls and potentially deeper reflectors. Moreover, the shear wave velocity of the material must be determined with high accuracy to reliably calibrate the depth scale of the reconstructed images.

Against this background, the present study pursues multiple objectives. Foremost, it aims to investigate the potential of UST in combination with FT-SAFT reconstruction for a complex measurement scenario within the KC. Particular emphasis is placed on comparing the different measurement configurations—an 8-channel UST device for rapid overview scans and a 16-channel UST device built out of two linked UST devices for high-resolution, detailed investigations—with respect to signal-to-noise ratio, penetration depth, image quality, and practical feasibility.

A further objective is to assess the validity of the systematic velocity calibration derived from surface-wave measurements on the 45 accessible blocks of the KC, with particular attention to the remaining depth uncertainties associated with establishing a chamber-wide representative shear wave velocity. The aim is to derive a three-dimensional (3D) model of the lower block rows from reconstructed two-dimensional (2D) images derived from measurement profiles, visualize block thicknesses and selected internal reflectors, and provide a basis for subsequent structural analyses or investigations of the construction method of the KC.

**Fig. 1:**
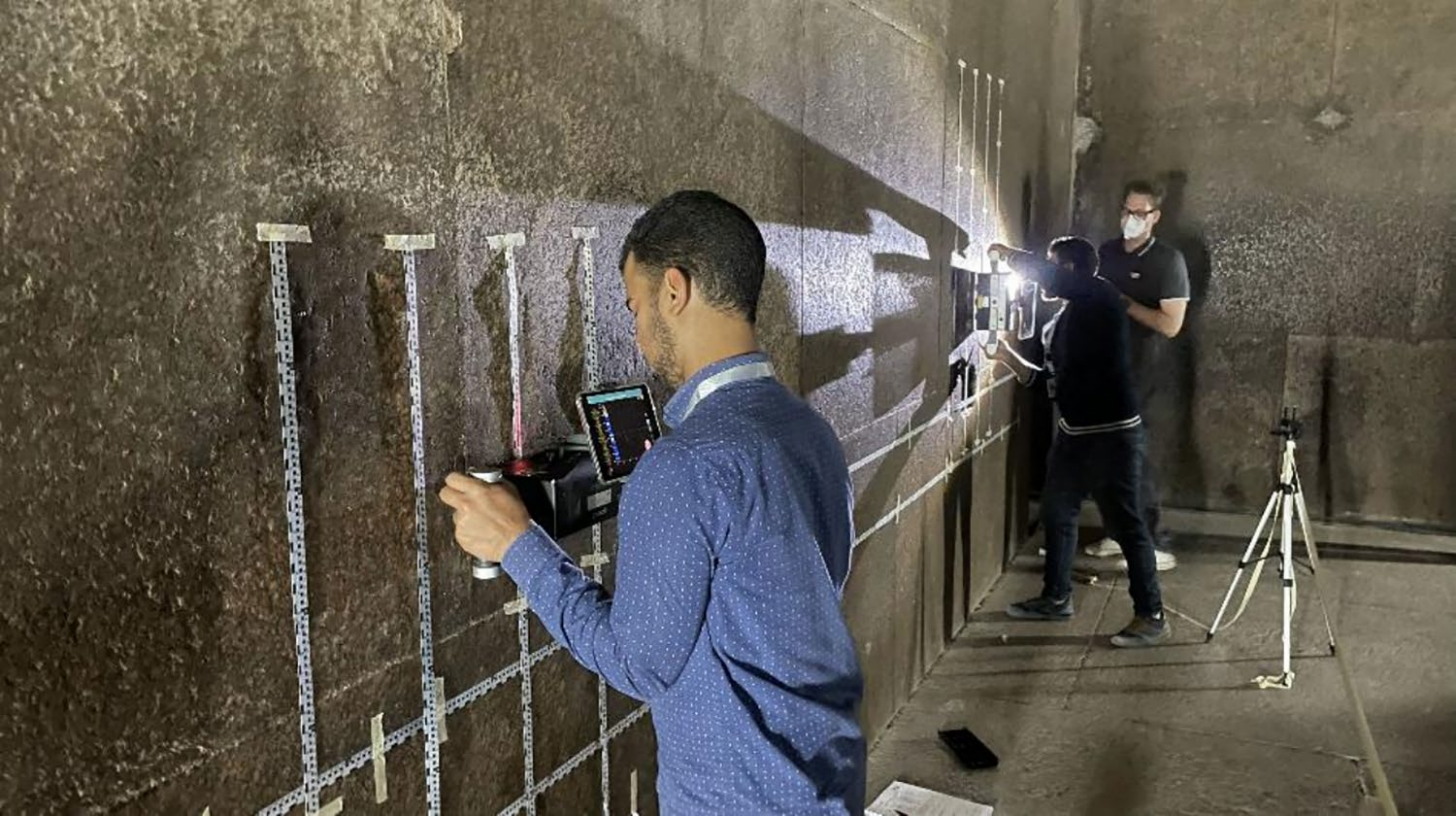
SP team members performing measurements on the KC north wall (NW) with two UST devices.

The measurements were conducted during the SP project measurement campaigns in February and October 2022. The investigation strategy initially involved linear surface scans with an 8-channel UST device to obtain a rapid overview of all accessible lower block rows along the walls of the KC and to identify areas with notable reflection patterns, as illustrated in Fig. 1. In a second step, selected profiles, particularly in the northwestern part of the chamber, were remeasured using two coupled UST devices in a 16-channel configuration to increase data density and improve image quality for deeper reflectors.

## Method and measurements

First, the approach chosen for the measurement campaigns will be examined in more detail, including the equipment and software used. In this context, the following sections outline the strategic considerations for selecting the block rows examined, describe the instruments used, and, finally, explain the measurement data collection. Data Processing shows how the collected data is converted into reconstructed images using the FT-SAFT algorithm,  while the section about the velocity calibration and validation explicitly describes how the correct depths can be represented in this process.

### Measurement strategy

**Fig. 2:**
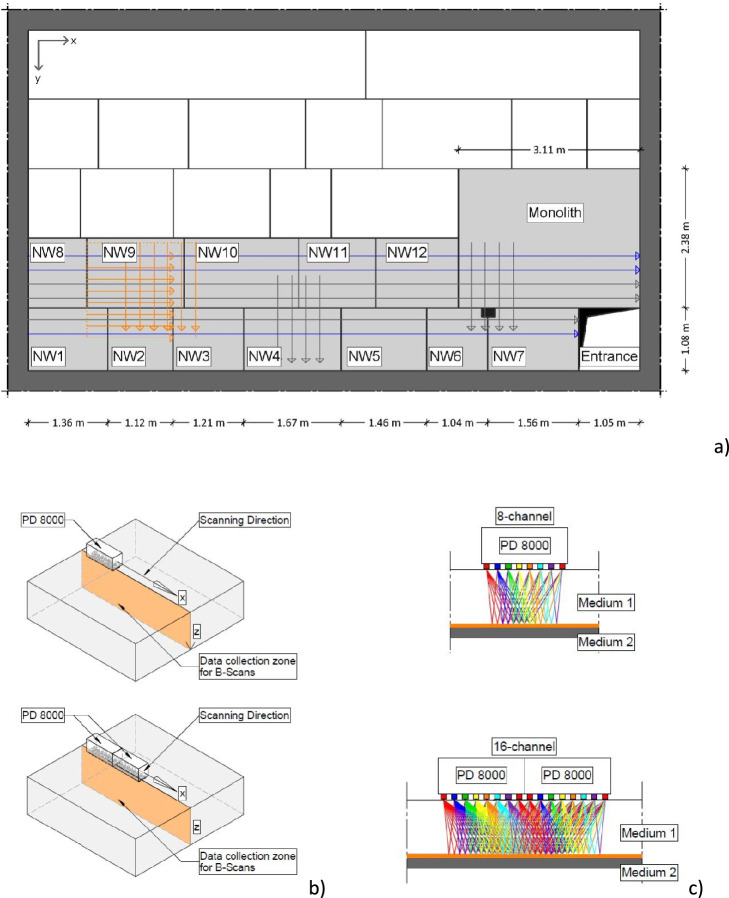
(**a**) Overview of the measurement profiles on the NW of the KC (looking north). Each arrow represents a measurement performed with arrays having either 8 channels (colored gray and blue) or 16 channels (colored orange). Image reconstruction was only done for the latter. “NW,” followed by a number, indicates the number used for each block. (**b**) Illustration of a line scan measurement (B-scan) on a flat surface of a block with an 8-channel and 16-channel setup. (**c**) Cycle of emitted waves schematically represented by direct ray paths using an 8-channel setup, resulting in 28 A-Scans, and a 16-channel setup, resulting in 120 A-Scans. Measurement profiles for all four walls of the KC are shown in Fig. 9 of the Appendix.

One of the goals of the SP project in 2022 was to investigate the KC using state-of-the-art ultrasound equipment and to establish advanced reconstruction and imaging techniques for data processing. For this purpose, the granite block structure was examined based on Dormion’s representations of the KC^2^. In February 2022, preliminary measurements were conducted on these blocks to identify which areas would be potentially interesting to perform higher-resolution measurements. Anomalies found on the north wall (NW) were then examined in detail in October 2022. An example of the measured profiles is shown in Fig. 2a for the NW of the KC and in the appendix for the whole KC. The collected data were processed after the measurement campaigns using the method described in data processing and assembled for the 3D model shown in the section results, which is a first representation of the interior of the KC building blocks.

Given the limited time available for measurements inside the KC, the efficiency of the February 2022 campaign could be significantly improved by utilizing the two available devices independently. To define the designated measurement area and establish a coordinate reference, a reflective measurement tape was installed. The length markings on this tape were detected by a camera integrated in the UST device and automatically positioned within the measurement profile. In the next step, two teams were formed to position the UST devices along the walls in horizontal and vertical lines, following the mounted tape, and record measurements with an offset of approximately 0.15 m at each position. During the second campaign in October 2022, measurements with the 16-channel UST device were conducted on block NW9 and on the KC NW to clarify the findings from February 2022. For comparison between the two UST device configurations, one measurement with the 16-channel UST device was performed at the same position where measurements with the 8-channel UST devices had been conducted.

### UST device

For the ultrasound measurements, a state-of-the-art UST device^15^ was used with the following construction details: 24 dry-point contact shear wave transducers (DPC) are arranged in a 3 × 8 matrix. Three transducers in each column operate simultaneously, forming an 8-channel UST device. The spacing between adjacent rows and columns of transducers is 25 and 30 mm, respectively. The DPCs are spring-mounted, allowing for efficient coupling on rough or uneven stone surfaces without the need for additional coupling agents. The spring-mounted tips introduce a predominantly tangential force component into the surface, thereby generating primarily horizontal shear waves (SH-waves) while significantly reducing the proportion of compressional waves. The directional excitation of SH-waves is achieved by orienting the transmitting elements along the block surface, resulting in dominant propagation paths within the plane between the rows of DPCs in the UST device. This configuration enhances the detection of shear wave reflections from interfaces and inhomogeneities, thereby increasing the method’s sensitivity to cracks and zones of reduced stiffness.

The DPCs can be operated within a frequency range of 25–65 kHz by adjusting the device’s analog setup specifications. However, the resonance frequency of the transducer is located at 44.9 kHz. In this study, a center frequency of 25 kHz was used. One measurement with an 8-channel UST device provides 28 individual reflection signals (A-Scans) at a single measurement position. At the available center frequency of 25 kHz and a recording length of 4000 µs, a measurement on the granite blocks can be completed in less than a minute. In this configuration, shear wave penetration depths of up to 3 m were achieved in the KC for the stony material. The manufacturer of the UST device states that the maximum penetration depth is 2 m, whereas other publications, such as Elkarmoty [7] and Helal [] , report penetration depths of up to 2.5 m for limestone and granite. Two of these UST devices can be combined into a 16-channel UST device physical attachment. The increased number of transmitter–receiver combinations to 16 enables the generation of 120 A-Scans per measurement, up from 28. The data density and resolution of the reconstructed images increase accordingly using the 16-channel UST device (Comparision of 8- vs. 16-channel). Figure 1 gives an impression of the ultrasound measurements at the KC NW. Illustrations of the ray paths of excited and reflected shear waves using the 8-channel and 16-channel UST devices are shown in Fig. 2b and Fig. 2c, respectively.

### Data acquisition and transducer characterization

During a measurement cycle, each column emits a pulse in turn, while the columns to the right of the transmitter act as receivers. Each measurement cycle involves sequential pulsing of the eight transmitter columns, with each column emitting a broadband shear wave pulse. The columns positioned to the right of the active transmitter simultaneously record the reflected signals. Pulse repetition is digitally controlled, with a recording window of 4000 µs per A-Scan, allowing complete capture of reflections from depths up to approximately 3 m in the granite.

To clarify how the acquired ultrasound data, in the form of A-Scans, appear, the frequency response function (FRF) needs to be specified. The manufacturer states that the DPC frequency peak lies close to 40 kHz^15^, however, independent measurements have found the center frequency to be approximately 10% higher ^16^. Anyhow, the frequency response function (FRF) of the shear wave DPC transducer is defined as the ratio of the complex spectra of the output and input signals. In general, the FRF formula of such ultrasound devices can be written as follows^17^:

$$H(f)=\frac{Y(f)}{X(f)}$$

where $X(f)$ is the Fourier transform of the excitation voltage signal and $Y(f)$ is the Fourier transform of the measured transducer response voltage.

For the laboratory-determined amplitude^16^ response, the measured spectrum was normalized to the maximum value at the resonance frequency (2) and expressed in decibels:

$${FRF}_{dB}(f)=20{\mathrm{l}\mathrm{o}\mathrm{g}}_{10}\left(\frac{|H(f)|}{|H({f}_{max})|}\right)$$

The FRF characterization reveals that the DPC transducer exhibits a distinct maximum at ${f}_{max}=44.9 kHz$ with a – 6 dB bandwidth of 20.1–60.8 kHz and a – 20 dB bandwidth of 4.6–83.4 kHz (Appendix, Fig. 16). This usable bandwidth provides both sufficient penetration depth and good temporal resolution for detecting reflected shear waves in granite. Combined with the transducer’s measured frequency response, the chosen excitation pulse with a center frequency of 25 kHz provides an effective operating range of 20.1–60.8 kHz, ensuring a favorable balance between penetration depth and lateral resolution in the granite blocks. A plot of the FRF characterization is shown in Appendix Fig 16. Shear wave velocities were measured at the surface of each of the 45 accessible granite blocks across the four walls (NW, EW, SW, WW) of the structure. In addition to the measurements intended only to provide an overview, shear wave velocities near the surface were determined on the 45 accessible granite blocks in the two reachable rows of the KC. The lower row of the NW contains blocks NW1, NW2, NW3, NW4, NW5, NW6, NW7, and the upper row contains NW8, NW9, NW10, NW11, NW12 and the Monolith; for the other rows of the east wall (EW), south wall (SW), and west wall (WW), the blocks are labeled analogously. To calibrate the shear wave velocity c_s_, the UST device was pressed on the surface, and multiple shear wave velocities were collected by emitting a pulse from the first DPC column. They were recorded by the next seven columns of built-in DPCs. The shear wave velocity ${c}_{s}$ is calculated from the measured travel time of a single pulse, using the different arrival times to the other DPC rows. From these measurements, local velocity ${c}_{s}$ values were computed and used to convert travel times to depth in the subsequent reconstructed images. Spatial variations in shear wave velocity were observed across different blocks, likely reflecting variations in weathering, microcracking, or granite composition. These local velocity estimates were incorporated into the depth conversion for each measurement position, thereby improving the accuracy of reflector depth interpretation. The complete velocity calibration results and validation are discussed in the section about velocity calibration and validation..

**Fig. 3:**
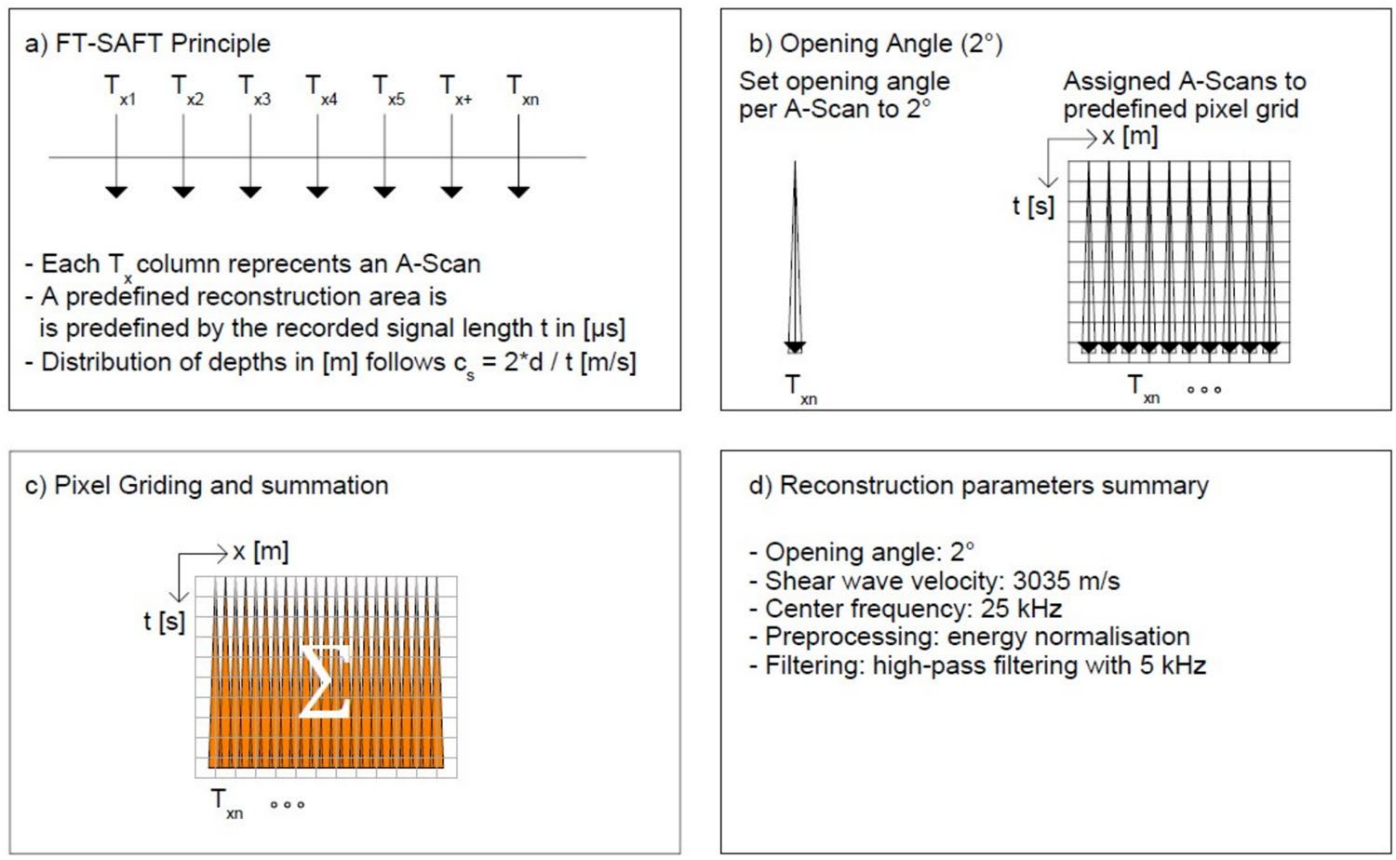
Processing steps with the 2D FT-SAFT reconstruction algorithm for the KC data. (**a**) FT-SAFT Principle. (**b**) Opening Angle application. (**c**) Pixel gridding and the summation process with the FT-SAFT-Algorithm. (**d**) Summary showing selected parameters used for reconstructing KC images.

### Data processing

The data collected during both measurement campaigns were reconstructed. The goal was to generate reconstructed 2D images of the data. For this purpose, the FT-SAFT reconstruction^13^ method was used. This approach had already been applied to the image reconstruction of the SP-NFC^7^ and the anomalies found on the Menkaure Pyramid’s east wall^9^. The following formulas describe, first, the SAFT-Algorithm in general and, second, the specific FT-SAFT formula^13^ used for the data obtained with the UST device.

For the general SAFT approach using single-element A-scans acquired at array positions ${\mathrm{r}}_{n}$, the reconstructed image intensity at image point $\mathrm{r}$ is given by:

$$I(\mathrm{r})=\sum_{n} {w}_{n}(\mathrm{r}) {s}_{n} (t={\tau}_{n}(\mathrm{r}))$$

with the travel time:

$${\tau}_{n}(\mathrm{r})=\frac{2}{{c}_{s}} \| \mathrm{r}-{\mathrm{r}}_{n}\|$$

where $I(\mathrm{r})$ is the reconstructed image, ${s}_{n}(t)$ is the recorded signal at array position $n$, ${\mathrm{r}}_{n}$ is the position of the respective array element, ${\tau}_{n}(\mathrm{r})$ is the two-way travel time from the array element to the image point and back, ${c}_{s}$ is the shear wave velocity, and ${w}_{n}(\mathrm{r})$ is an optional weighting or normalization factor. Instead of a Total Focusing Method (TFM) reconstruction with Full Matrix Capture (FMC) data, a frequency-domain implementation of SAFT (FT-SAFT) is used in this study because the employed UST device captures data in half-matrix schemes rather than complete FMC datasets^18^. In InterSAFT, a software module inside the Software Pundit_Vision^19^, both SAFT and FT-SAFT are available as reconstruction methods, and linear-array data are processed with a dedicated reconstruction function^13^. For each array element at position ${\mathrm{r}}_{n}$, the recorded time signal is transformed into the frequency domain as:

$${S}_{n}(\omega )=\mathcal{F}\{{s}_{n}(t)\}={\int}_{-\infty }^{\infty } {s}_{n}(t) {e}^{-\mathrm{i}\omega t} \mathrm{d}t$$

where $\omega$ denotes the angular frequency. The image is then obtained by coherent summation over the array elements and over the relevant frequency band $[{\omega}_{\mathrm{m}\mathrm{i}\mathrm{n}},{\omega}_{\mathrm{m}\mathrm{a}\mathrm{x}}]$:

$$I(\mathrm{r})=\sum_{n} {\int}_{{\omega}_{\mathrm{m}\mathrm{i}\mathrm{n}}}^{{\omega}_{\mathrm{m}\mathrm{a}\mathrm{x}}} {W}_{n}(\mathrm{r},\omega ) {S}_{n}(\omega ) \mathrm{e}\mathrm{x}\mathrm{p} (\mathrm{i}\omega {\tau}_{n}(\mathrm{r})) \mathrm{d}\omega$$

The term ${W}_{n}(\mathrm{r},\omega )$ represents an optional weighting function, which can account for aperture tapering, geometric spreading, or frequency-dependent amplitude corrections. In practical terms, the opening-angle setting used in InterSAFT can be represented within this weighting term by assigning zero contribution outside the selected aperture and a finite taper inside the selected aperture^13^. This frequency-domain formulation allows explicit control over the effective bandwidth used for reconstruction and can be advantageous when analyzing the influence of filtering and frequency content on image resolution and penetration depth.

Within this FT-SAFT method, each recorded value in the individual A-scans is mapped to a predefined sample length and assigned to pixels (2D) or voxels (3D), which form the reconstruction grid. The overlapping signal contributions are summed coherently in the Fourier domain, while the effective aperture can be restricted by a defined opening angle^13^. This produces the reconstructed images.

This reconstruction technique significantly enhances the visibility of various reflections by reducing the influence of side-wall reflections (reflections from block joints)^12^^[,20^. Figure 3 provides an overview of the 2D FT-SAFT principle, the assignment of A-Scans, and the application of the opening angle for summation and superposition of the data within the predefined evaluation plane and corresponding pixels. First, the collected data, in the form of A-Scans, is converted to a format that the reconstruction software Pundit_Vision^19^ can work with. After reading the data, the software creates a pixel grid with the length of the profile and the number of A-Scans per profile. Afterward, the time or depth axis (labeled with t in [s]) is derived from the data samples (here 4000 µs) in one A-Scan shown in Fig. 3a. After that, the A-Scans are assigned to this pixel grid at their location in the profile in the x-axis [m]. Then, the A-Scans are overlayed with an opening angle, which leads to an overlap of neighboring A-Scans, which is shown in Fig. 3b. The usage of narrow opening angles reduces sidewall and joint reflections, enhancing the visibility of internal reflectors, consistent with the reconstruction settings applied in prior SP investigations (e.g., SP-NFC^7^). The 25 kHz frequency is listed in Fig. 3d because it is originally an acquisition parameter, but it is also provided to the software for preprocessing and filter options. After that, the FT-SAFT algorithm sums the amplitude values of the A-Scans across the entire pixel grid, as described above. This is shown in Fig. 3c. Additional reconstruction parameters are shown in Fig. 3d. The result of the whole reconstruction process is a reconstructed image in a grayscale color map.

The following list gives an overview of the most important reconstruction steps used with the Pundit_Vision software package^19^:

#### Preprocessing and artifact suppression 

Before reconstruction, the obtained data were preprocessed to suppress overshooting amplitude values, thereby reducing typical artifacts, such as half-circle-shaped overshooting reflector patterns (tornado patterns), which ultimately disturb the resulting 2D images. The following steps were applied:

Energy normalization: The process of normalizing individual A-scans leads to a reduction in the variability of the maximum amplitudes across different transducers. This, in turn, results in a decrease in the difference in energy recorded by each transducer (e.g., due to a difference in its sensitivity or coupling) and a concomitant reduction in spurious overshoots.

#### Postprocessing filtering and artifact suppression

After preprocessing, filtering steps were applied to remove low-frequency noise acquired by the measurements inside the chamber due to noise, working with two devices, and visitors could be emitting different SH-waves at very low frequency.

The raw signals undergo high-pass filtering at 5 kHz to remove low-frequency ambient noise and structural vibrations that could interfere with the shear wave arrivals. This cutoff frequency was selected to preserve the full effective bandwidth of the transducers (as shown in Fig. 16) while suppressing unwanted low-frequency components. Given the −20 dB bandwidth extending down to 4.6 kHz, the 5 kHz high-pass filter introduces minimal attenuation to the usable signal content while effectively eliminating noise below the transducer’s operating range.

#### Limitations

Because all measurements were performed at a center frequency of 25 kHz, the following physical effects are inherent:

- Mode-converted longitudinal waves may appear in some recordings
- Residual half-cycle artifacts may persist even after filtering, due to limitations in the filtering process or different higher frequencies above the 5 kHz frequency.
- Slight variations in block properties (e.g., density, Poisson’s ratio) may introduce small depth uncertainties, which are addressed in velocity calibration and validation.

These limitations do not affect the identification of major reflectors but should be considered when interpreting weaker reflectors.

### Velocity calibration and validation

For the FT-SAFT reconstruction, it is essential to determine an optimal shear wave velocity (${c}_{s}$), since the measurement data are initially A-Scans that are mapped into a spatial image. The A-Scans hold only the measured runtime and amplitude values. From the measured time, the correct depth can be recalculated using the shear wave velocity. So the distance traveled—and thus the depth of a reflector—can only be represented accurately when the shear wave velocity of the granite blocks of the KC is known. The shear wave velocities of the 45 blocks on which the study was performed were measured with the UST device^15^. The results of these measurements are presented in Table 1 for the lower and upper block rows of the KC.

**Table 1:** Shear wave velocities derived from surface measurements on the lower and upper block rows of the KC.

Block row	Surface wave velocity mean values in [m/s]	Standard deviation in [m/s]	Coefficient of variation (CV) in [%]	Number of blocks in each row
NW lower	3063	73	2.38	7
NW upper	3035	37	1.22	4
EW lower	3067	101	3.29	4
EW upper	3051	25	0.82	3
SW lower	3039	128	4.21	10
SW upper	3054	93	3.05	8
WW lower	2948	46	1.56	5
WW upper	3006	75	2.50	4

Across all measured blocks, the chamber-wide mean shear wave velocity (${c}_{s, KC}$) is 3035 m/s with a standard deviation of 90 m/s. This chamber-wide mean value was therefore adopted as the representative reconstruction velocity for all reconstructed images. Assuming a fixed two-way travel time, the relative depth uncertainty scales with the relative uncertainty of the velocity $(\Delta z/z\approx \Delta c/c$). With a standard deviation of 90 m/s relative to the mean value of 3035 m/s, the corresponding coefficient of variation (CV) of the velocity is approximately 3%. Even under the conservative assumption of a coefficient of variation (CV) of ± 5% for (${c}_{s, KC}$), the resulting depth uncertainty remains below ± 70 mm. The calculation for the thickness determination is done with the following two calculations:

$${c}_{s,KC}= \frac{2d}{t}$$

With: c_s,KC_ = shear wave velocity measured for the block rows in [m/s], d = depth in [m], and t is time in [µs]

$$d= \frac{{c}_{s,KC}*t}{2}$$

With: c_s,KC_ = shear wave velocity measured for the block rows in [m/s], d = depth in [m], and t is time in [µs].

To independently validate the depth scale of the FT-SAFT reconstructions, comparative measurements were conducted on the large monolithic block located above the entrance of the KC (Fig. 2a), which is also made of granite and accessible from two sides. The physical depth of the monolith could therefore be measured not only with the UST device but also with a laser distance meter. This allowed for a comparison of the measured physical width with the depth of the backwall reflection in the FT-SAFT reconstruction. The reconstructed backwall appears at approximately 1.34 m, which matches the in-situ measurement within a tolerance of ± 30 mm. This is consistent with the theoretical uncertainty derived from the velocity statistics.

Overall, the velocity calibration and validation show that ${c}_{s, KC}$ of 3035 m/s is sufficiently accurate to identify subsurface reflectors at their correct depths. Although individual blocks may exhibit slight variations in material properties (e.g., density or Poisson’s ratio), these do not compromise the robustness of the reconstruction. Therefore, the single representative velocity was applied to all ultrasonic datasets acquired in the KC, due to the limitation that a comparison between a physical depth and the depth measured with the UST is only feasible on the monolith above the entrance.

## Results

To provide an overview of the KC, the images reconstructed from the 8-channel UST device are evaluated first. Subsequently, measurements from the 16-channel UST device are analyzed to present the results for the specific area at the northwestern corner of the KC. This is followed by a comparison of the 8-channel and 16-channel UST devices based on the measurement results, before the data are integrated into a 3D interpretation and presented as a comprehensive overview of the KC. It should be emphasized that the depths of the granite blocks are determined from reflections observed in the reconstructed images. An overview of the most common interpretations of reflectors identified in similar stone blocks has been published in Elkarmoty^7^ and Helal^14^. For concrete, relevant interpretations of reflections, see Mayer^20^, Rabe^12^, and Maier^21^. In the reconstructed images presented here (Fig. 3 b), hyperbolic reflections, for example, can be seen, indicating the rear corners of the blocks. Linear reflections with an inclination of approximately 45° originating from the measurement surface are associated with joints between blocks or cracks within the blocks. In some blocks, a backwall echo can be identified. While these reflector types provide useful indicators of internal geometry, their interpretation in heterogeneous granite remains non-unique, because multiple scattering and grain-scale heterogeneities can produce similar signatures.

### Results of the 8-channel measurements

**Fig. 4:**
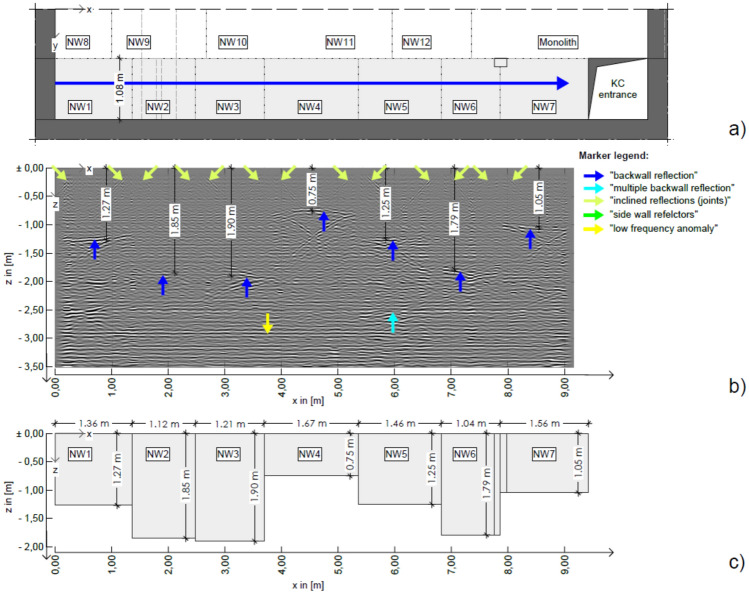
Reconstructed image and extracted block depth information from a measurement profile conducted on the lower blocks of the KC NW. The measurements were carried out using an 8-channel UST device. (**a**) Measurement positions along the NW. (**b**) Reconstructed image with highlighted reflections (in yellow). (**c**) Plan view of the interpretation and derivation of block depths and widths based on the reconstructed image.

Images reconstructed from measurements with the 8-channel UST device showed reflections on all walls of the KC. While the remaining evaluations are provided in the Appendix, Fig. 4 presents the NW of the KC, as the reflections are particularly well visible there. Figure 4a illustrates the measurement profile, whereas Fig. 4b displays the reconstructed images, and the interpretation is graphically represented in Fig. 4c. A top view is provided along with the measurement location evaluated for the NW of the KC in the lowest accessible block section.

In the NW view of the KC (Fig. 4a), which includes the numbered blocks, a blue arrow indicates the measurement profile. This line scan, performed approximately in the middle of the block row, begins about 10 cm from the corner due to the size of the measurement device and ends shortly before the KC entrance. From the data acquired along this line, the reconstructed FT-SAFT images (Fig. 4b) were generated, with the relevant reflections marked by arrows. The blue arrow-shaped markers indicate the reflections that are linked to the backwall of each block, with the associated depth indicated by a scale bar. However, in a heterogeneous material like granite, these reflections could also originate from inclusions or other shell-like internal features within the granite. The yellow arrow-shaped markers indicate reflections from the joints between the granite blocks, which are also linked to the side-wall echoes of the blocks. These reflections are inclined at approximately 45° to the accessible surface of the block. This inclination is expected only when the blocks are rectangular-shaped and allows conclusions to be drawn regarding the lateral extent of the blocks at the respective joints. Figure 4c presents these results in a graphical representation that provides a more intuitive visualization and serves as preparation for the 3D model. A tabulated list of the various identified block depths for all investigated blocks, organized by wall, is provided in the Appendix, Figs 9 to 15 and table 3.

### Results of the 16-channel measurements

While searching for areas of interest during the measurements with the 8-channel UST device, block NW9 is noteworthy. A hyperbolic reflection—albeit only faintly visible—can be observed at a depth of approximately z = − 2.0 m. For this reason, this region was also examined in greater detail using the 16-channel UST device. In Fig. 2a, the conducted measurements on the surface of block NW9 are shown as orange arrows. 

**Fig. 5:**
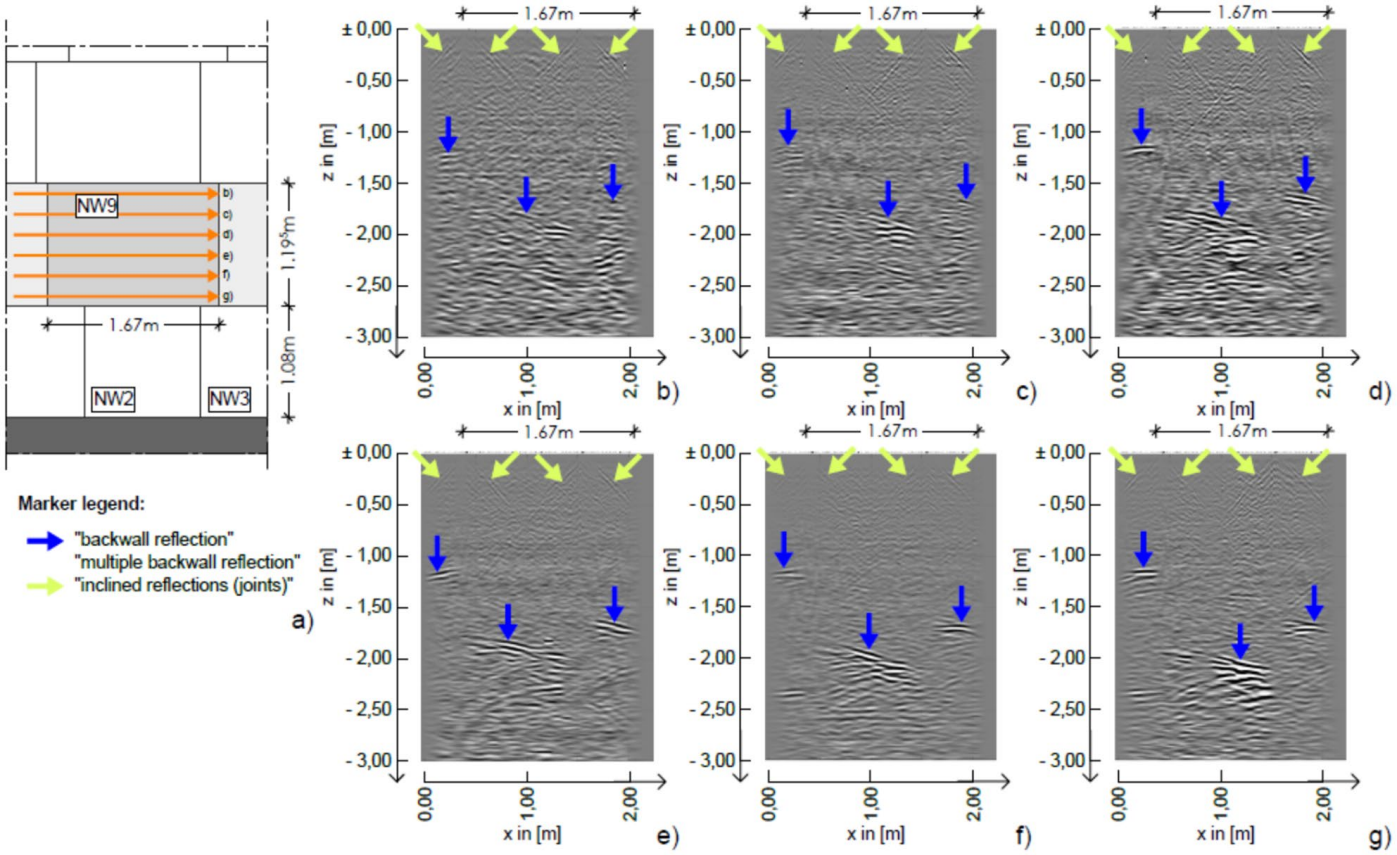
Reconstructed images from the measurements with the 16-channel UST device on Block NW 9 to clarify the reflections behind Block NW 9. (**a**) Measurement locations on block NW9. (b) Reconstructed image at the top. (**g**) Lowest line scan close to the bottom of the block. Images (b) to (g) are also marked in (a).

The data of NW9 is displayed as reconstructed images in Fig. 5. Hyperbolic reflections, indicating an anomaly, are highlighted with blue arrow-shaped markers in each image. The figures show the reflections ranging from those recorded near the upper edge of the block (Fig. 5a) to those obtained at the lower edge (Fig. 5g). The anomaly is visible in each reconstructed image and thus spans the entire height of the block. As shown in Fig. 5, it is located at approximately z = − 2.0 m and therefore appears to lie behind the block itself or to be part of the backwall of block NW9. In each reconstructed image, additional reflection layers at approximately z = − 2.0 m are visible in the center. Moreover, inclined reflections at roughly 45° from the surface are visible. These are associated with surface discontinuities, such as cracks within the block and the two adjacent block joints. Nevertheless, they indicate a rectangular geometry of the granite block NW9.

**Fig. 6:**
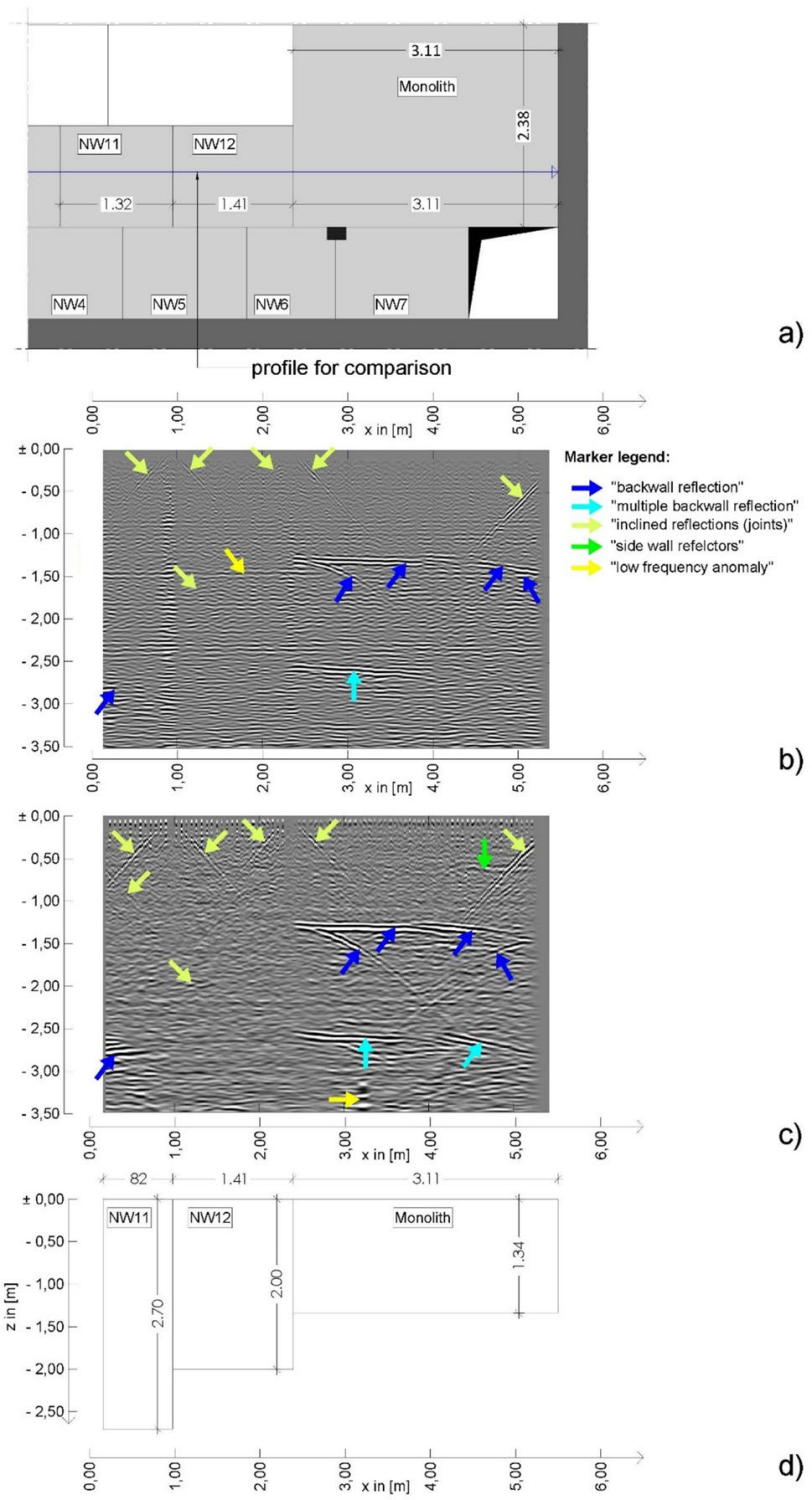
(**a**) View from the northwest showing the position of the profile used for comparing the measurements. (**b**) Reconstructed image from a data set collected with the 8-channel UST device. (**c**) Reconstructed image from a data set collected with the 16-channel UST device. (**d**) Interpretation and derivation of the block sizes from the reconstructed data.

Compared to the 8-channel configuration, the 16-channel measurements provide a higher data density and an improved signal-to-noise ratio, resulting in sharper backwall echoes and a clearer representation of the hyperbolic reflectors. An interpretation of this reflection located behind the backwall of block NW9 is discussed later..

However, given the limited aperture and the complex wave propagation in massive granite blocks, this hyperbolic reflector should be regarded as a plausible but not uniquely constrained indication of an internal boundary or cavity.

### Comparison of 8 vs. 16 channels

To explore the potential of the different configurations, a supplementary study was conducted to compare the reconstructed images obtained with the 8-channel and 16-channel UST devices. Particular attention was given to the respective increased penetration depth and the level of detail in the deeper recorded regions. 

The reconstructed images from these two measurements now allow evaluation of the hypothesis that a measurement with the 16-channel UST device provides a higher signal-to-noise ratio (SNR) than one with the 8-channel UST device. This is assessed, on the one hand, by evaluating the visibility of individual reflectors and, on the other hand, by examining the number of A-Scans recorded at a given measurement position. A comparison of the reconstructed images from both configurations is shown in Fig. 5 for the same measurement line on KC NW.

The reconstructed image in Fig. 5c, based on the 16-channel UST device, exhibits clearly more pronounced reflections of the same anomaly at a depth of z = − 2.7 m, as well as sharper details of the backwall and the inclined reflections, compared to the reconstructed image based on the 8-channel UST device in Fig. 5b. A hyperbolic reflection and the second backwall reflection of the monolith above the entrance (marked with blue arrows inside Fig. 6b,c) likewise confirm this result, as does a small reflection at a depth of z = − 0.7 m on the right side of the reconstructed image (marked with a green arrow inside Fig. 5b,c). 

With respect to the quantity of A-Scans generated, the 16-channel UST device provides 120 A-Scans across an effective device length of (16 − 1) × 0.03 m = 0.45 m, while the 8-channel UST device, with an effective device length of (8 − 1) × 0.03 m = 0.21 m, produces only 28 A-Scans. This means that—even if measurements with the 8-channel UST device are repeated three times with an overlap of 0.15 m in the same area—only 84 A-Scans can be obtained over the same measurement distance. The 16-channel UST device, therefore, provides higher data density with less effort, albeit at the cost of more challenging handling and increased data storage requirements. The amount of data increases from 2,5 MB for a single 8-channel acquisition to up to 5 MB for a single acquisition using the 16-channel UST device.

At first glance, the image reconstructed from the 16-channel UST device appears visually clearer and less affected by background noise than that from the 8-channel UST device, suggesting an improved SNR. Since such visual impressions can be subjective, a quantitative comparison of the reconstructed images is required to substantiate this observation. 

To quantitatively compare the data quality of the two acquisition configurations, an A-scan-based SNR analysis was performed on two measurement lines recorded at the same position, one using the 8-channel setup and the other using the 16-channel setup. For each A-scan, the noise level was estimated from the first 10% of the recorded time samples (pre-arrival window), and the signal amplitude was defined as the maximum absolute amplitude over the full time window. The SNR was then computed as^22^:

$${\mathrm{S}\mathrm{N}\mathrm{R}}_{i}=20{\mathrm{l}\mathrm{o}\mathrm{g}}_{10}({A}_{\mathrm{m}\mathrm{a}\mathrm{x},i}/{\sigma}_{\mathrm{n}\mathrm{o}\mathrm{i}\mathrm{s}\mathrm{e},i})in decibels [dB].$$

**Fig. 7:**
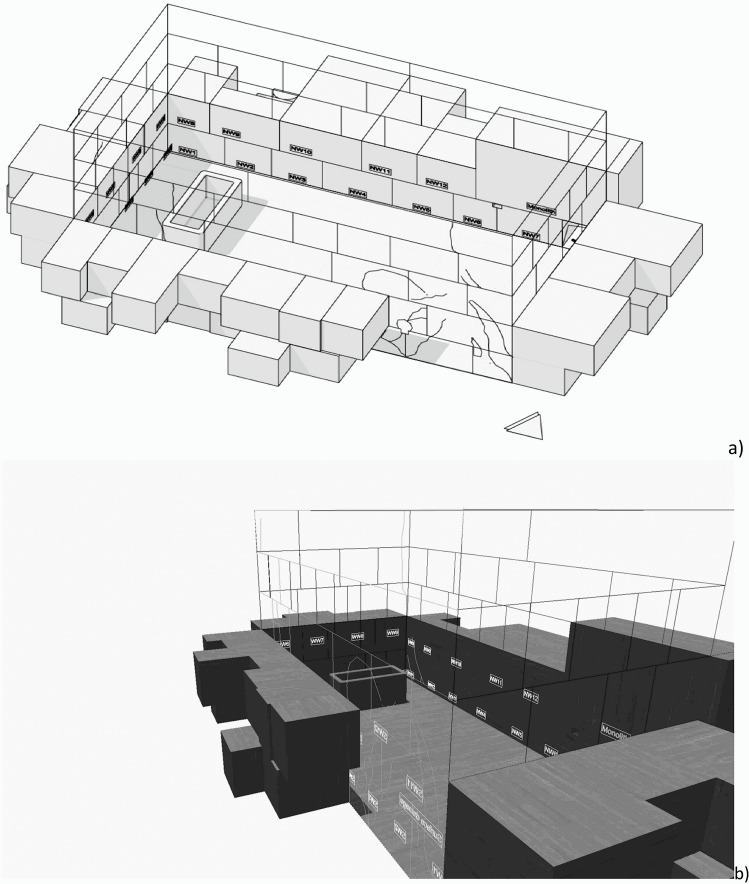
3D model of the KC with the measured sizes of the blocks. (**a**) Chamber view from the northwest with an arrow indicating the north direction. (**b**) Animated GIF of the entire chamber. Both representations show the sarcophagus in the western center of the chamber and the monolith block above the entrance; the positions of the blocks are labeled.

The 16-channel line (6480 A-scans) exhibited a mean SNR of 13.6 dB with a standard deviation of 3.69 dB, while the 8-channel line (2268 A-scans) yielded a mean SNR of 12.7 dB with a standard deviation of 3.01 dB. The minimum and maximum SNR values were 7.42 dB and 45.4 dB for the 16-channel configuration and 8.65 dB and 50.9 dB for the 8-channel configuration. Overall, the 16-channel configuration provides a moderately higher average SNR, whereas both configurations show comparable spread and similar peak SNR values along the measurement line. Table 2 summarizes the A-scan-based SNR statistics for the 8-channel and 16-channel measurement lines compared in Fig. 7.

**Table 2:** A-Scan-based SNR statistics for 8-channel and 16-channel measurement lines compared in Fig. 6.

Metric	16-channel line scan	8-channel line scan
Number of A-Scans	6480	2268
MEAN SNR [dB]	13.6	12.7
Standard SNR [dB]	3.69	3.01
Minimum SNR [dB]	7.42	8.65
Maximum SNR [dB]	45.4	50.9

Using this approach, the results from the 16-channel UST device exhibit a higher SNR than those from the 8-channel UST device. This improvement is physically consistent with the increased number of independent channels, which enhances coherent signal contributions while reducing incoherent noise through averaging. These findings suggest that, for comparable acquisition times, 16-channel measurements are particularly advantageous for resolving deeper reflectors or small-amplitude anomalies reliably in historic stone masonry.

### Derivation of the 3D block model

**Fig. 8:**
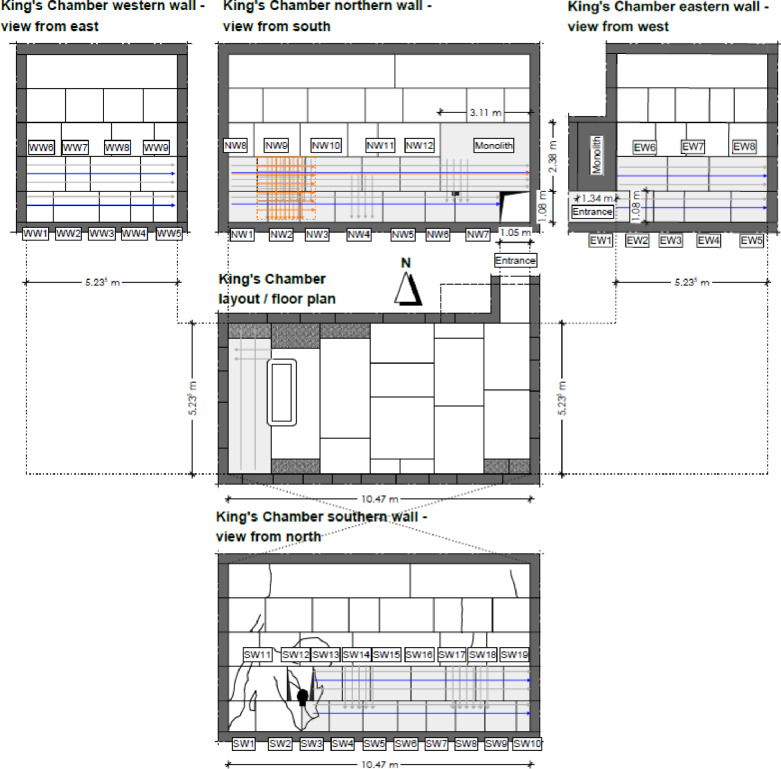
Drawings of King’s Chamber floor and walls with all measurement profiles scanned during the campaigns. The measurements where reconstruction and data evaluations were performed are colored blue and orange. Each arrow represents a measurement profile, with reconstructed images from the 8- and 16-channel UST device measurements colored in blue and orange, respectively.

**Fig. 9:**
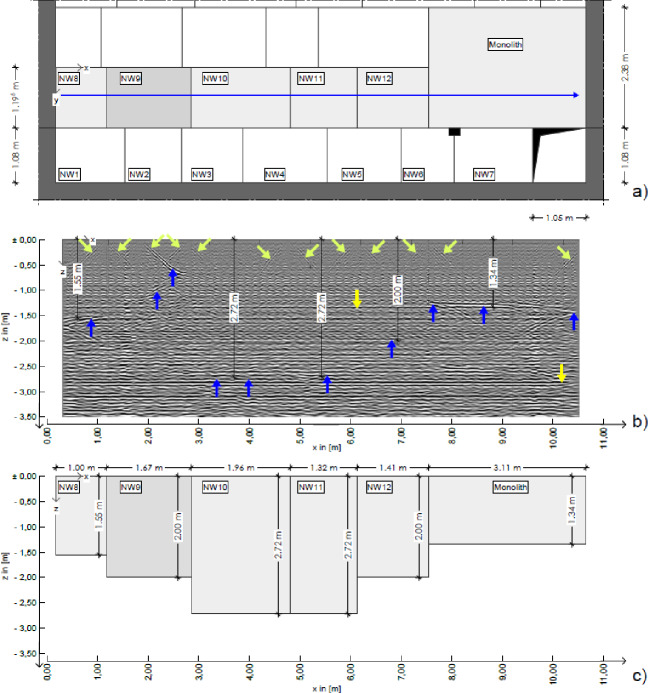
Reconstructed image and extracted block depth information from a measurement profile conducted on the lower blocks of the KC NW. The measurements were carried out using an 8-channel UST device. (**a**) Measurement positions along the NW. (**b**) Reconstructed image with highlighted reflections (in yellow). (**c**) Plan view of the interpretation and derivation of block depths based on the reconstructed image.

**Fig. 10:**
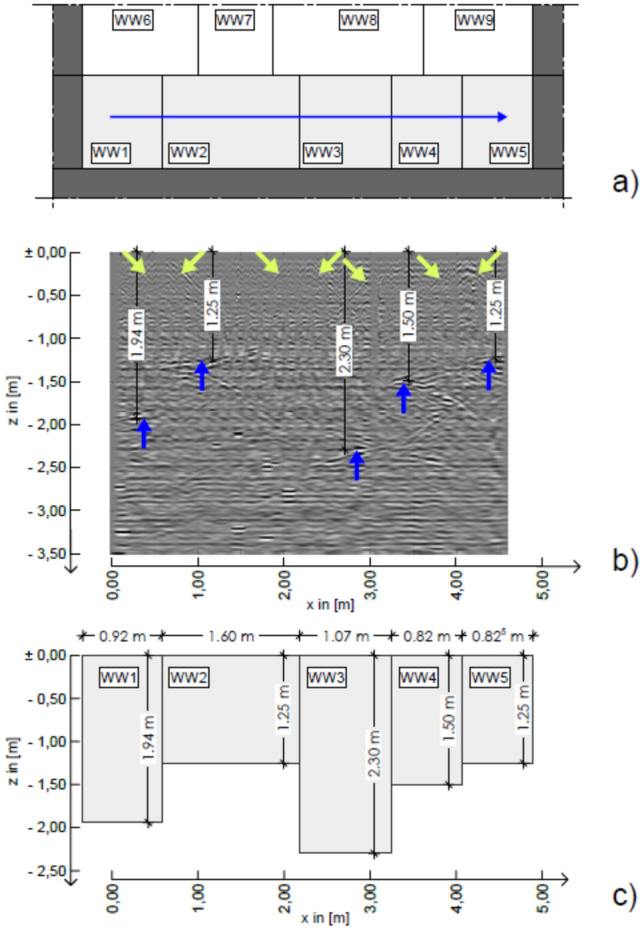
Reconstructed image and extracted block depth information from a measurement profile conducted on the lower blocks of the KC WW. The measurements were carried out using an 8-channel UST device. (**a**). Measurement positions along the NW. (**b**) Reconstructed image with highlighted reflections (in yellow). (**c**) Plan view of the interpretation and derivation of block depths and widths based on the reconstructed image.

**Fig. 11:**
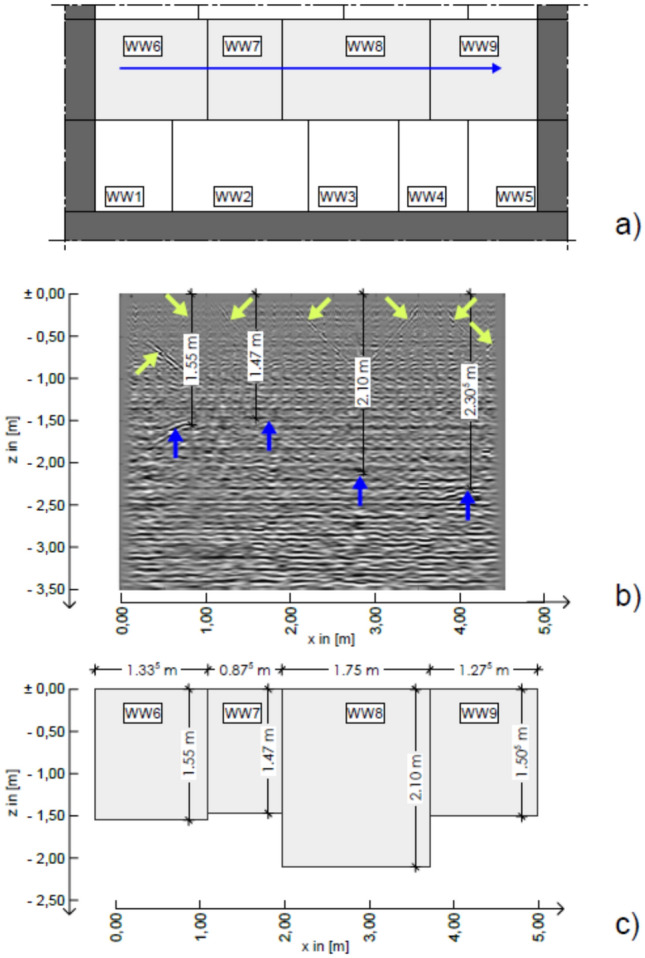
Reconstructed image and extracted block depth information from a measurement profile conducted on the upper blocks of the KC WW. The measurements were carried out using an 8-channel UST device. (**a**) Measurement positions along the NW. (**b**) Reconstructed image with highlighted reflections (in yellow). (**c**) Plan view of the interpretation and derivation of block depths and widths based on the reconstructed image.

**Fig. 12:**
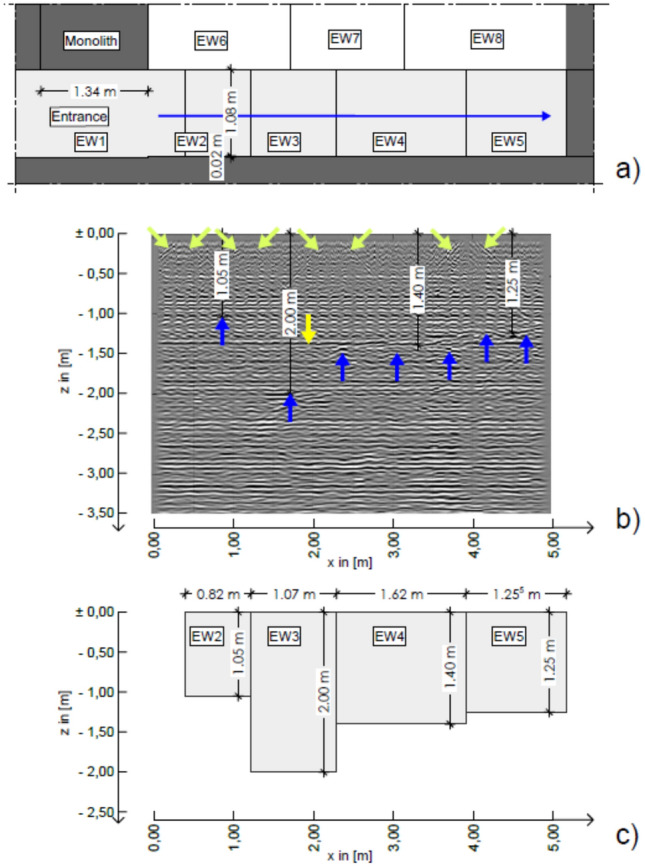
Reconstructed image and extracted block depth information from a measurement profile conducted on the upper blocks of the KC WW. The measurements were carried out using an 8-channel UST device. (**a**) Measurement positions along the NW. (**b**) Reconstructed image with highlighted reflections (in yellow). (**c**) Plan view of the interpretation and derivation of block depths and widths based on the reconstructed image.

**Fig. 13:**
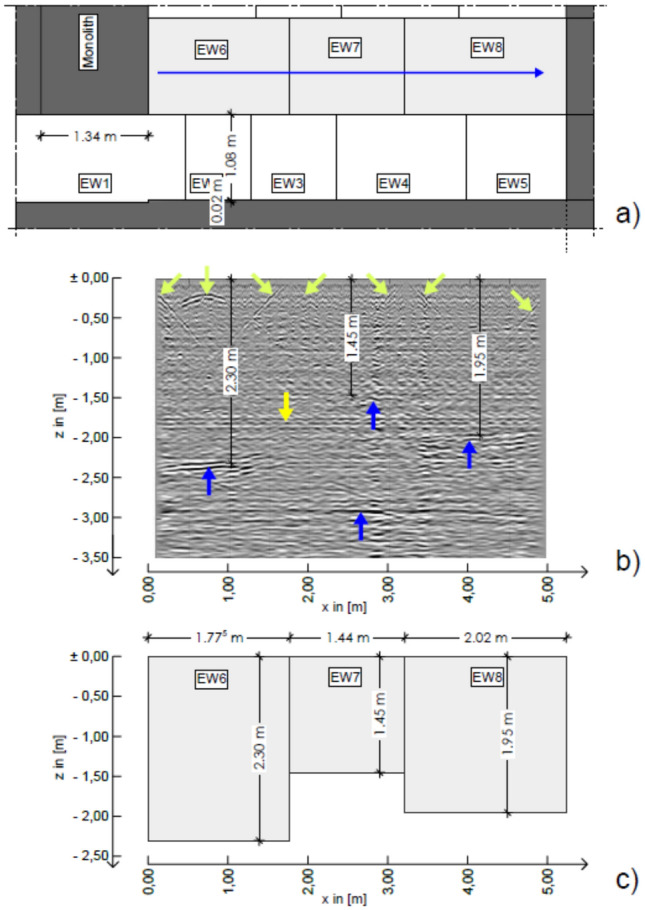
Reconstructed image and extracted block depth information from a measurement profile conducted on the upper blocks of the KC EW. The measurements were carried out using an 8-channel UST device. (**a**) Measurement positions along the NW. (**b**) Reconstructed image with highlighted reflections (in yellow). (**c**) Plan view of the interpretation and derivation of block depths and widths based on the reconstructed image.

**Fig. 14:**
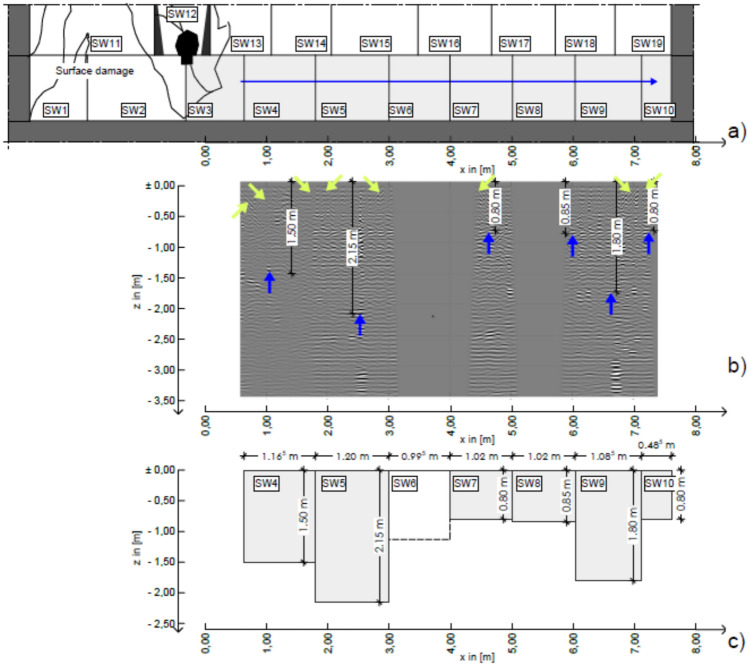
Reconstructed image and extracted block depth information from a measurement profile conducted on the lower blocks of the KC SW. The measurements were carried out using an 8-channel UST device. (**a**) Measurement positions along the NW. (**b**) Reconstructed image with highlighted reflections (in yellow). (**c**) Plan view of the interpretation and derivation of block depths and widths based on the reconstructed image.

**Fig. 15:**
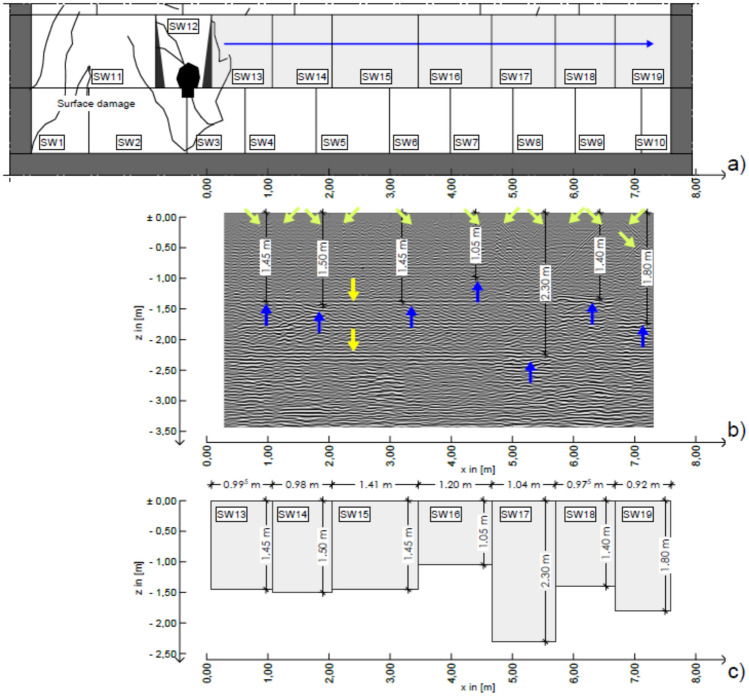
Reconstructed image and extracted block depth information from a measurement profile conducted on the upper blocks of the KC SW. The measurements were carried out using an 8-channel UST device. (**a**) Measurement positions along the NW. (**b**) Reconstructed image with highlighted reflections (in yellow). (**c**) Plan view of the interpretation and derivation of block depths and widths based on the reconstructed image.

**Fig. 16:**
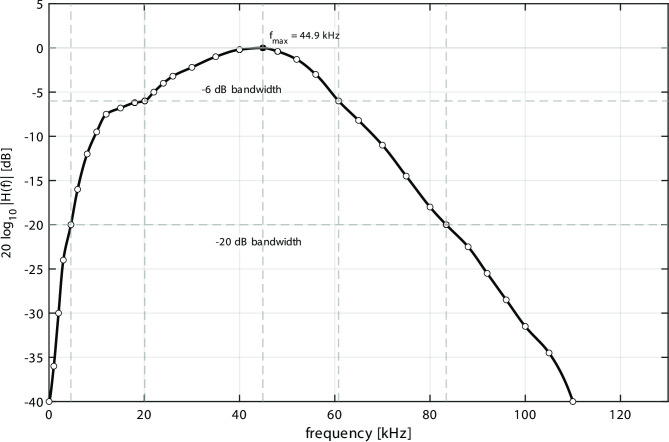
Frequency response function (FRF) of the DPC shear wave transducer showing normalized voltage in dB versus frequency. The peak frequency is 44.9 kHz, with a -6 dB bandwidth spanning 20.1–60.8 kHz and a-20 dB bandwidth spanning 4.6–83.4 kHz.

Based on the reconstruction of all measurements, a 3D model of the lower two block rows was created (Fig. 8), which can be used, e.g., by Egyptologists, for evaluating the construction history. The 3D interpretation of the data was performed using a CAD program, based on the two-dimensional drawings of the walls and, as previously explained, the reconstructed images (see Fig. 5 and Fig 9–15 in the Appendix). This representation depicts the various blocks as 3D bodies in gray, modeled as rectangular prisms with different widths and depths derived from the UST measurements. Fig. 8a visualizes the described backwall echoes for the surveyed blocks, while Fig. 8b is an animated GIF that provides a better overview of the blocks and their relative positions.

However, the 3D model still contains gaps in areas where block discontinuities identified in the evaluated data could not be determined precisely. For the same reason, the anomalies behind the blocks and behind NW9 are not shown; they are marked with a red circle to indicate their approximate position. Nevertheless, the 3D model provides a previously unavailable view of the interior of the block rows and demonstrates how FT-SAFT–based thickness estimates from 2D profiles can be integrated into a volumetric representation useful for structural assessment and archaeological interpretation.

## Discussion

The measurement strategy described in above proved to be a suitable approach. In the case of the KC, it was impermissible to achieve the research objective using invasive methods, particularly because it was not possible beforehand to assess whether and where such methods would be useful. The use of the 8-channel UST device and its extension to a 16-channel UST device was therefore an appropriate choice for non-destructively characterizing the dimensions of the granite block rows and particularly their depth. Dividing the campaign into an initial application of the 8-channel UST device for broad-area exploration and a following application of the 16-channel UST device for detailed investigation of regions of interest was efficient. It allowed the use of the available time frame for measurements, considering the complex boundary conditions of the touristed heritage site. 

The 8-channel UST device turned out to be a useful tool for “screening” large wall areas for voids, thickness of blocks, and geometric values. Locations with pronounced backwall reflections, locally increased reflector amplitudes, or irregular reflector geometries could be identified as potential anomalies. Measurements using the 16-channel UST device confirmed and refined these results (Sects. 3.2 and 3.3). By more than quadrupling the number of A-Scans per measurement position from 28 (8-channel UST device) to 120 (16-channel UST device), the effective aperture and data density increased substantially. This led to improved SNR, sharper backwall reflections, and more pronounced hyperbolic and inclined reflectors, thereby improving the detectability of deeper structures. Better data quality was particularly important for interpreting the reflectors behind block NW9, which appeared consistently in multiple reconstructed profiles and likely represent inclined or irregular internal boundaries within the KC’s structure rather than imaging artifacts. Their recurrence in independent measurements supports this interpretation, although multiple reflections and scattering in the granite cannot be fully excluded. The combination of both UST devices thus yields complementary data, with broad-area exploration complemented by high-resolution analysis, and provides a practical strategy that can be transferred to similar investigations on other large masonry structures.

Despite the complexity of the measurement conditions characterized by humidity, salt deposits, and pronounced surface roughness, many artifacts were successfully mitigated through the preprocessing and reconstruction procedures applied. Energy normalization and high-pass filtering at 5 kHz suppressed low-frequency noise (“tornado patterns”) and coupling-related artifacts, which occurred more frequently in the complex measurement environment, while preserving the effective bandwidth of the DPC transducers and thus maintaining a suitable compromise between penetration depth and temporal resolution. The chamber-wide mean shear wave velocity of 3035 m/s, derived from surface-wave measurements on 45 granite blocks, combined with the validation on the monolithic block above the entrance, allowed a depth scale with uncertainties of only a few centimeters. Based on the effective bandwidth of 20.1–60.8 kHz and the calibrated shear wave velocities, the achievable vertical resolution is on the order of a few wavelengths, i.e., several centimeters in granite, which is consistent with previous SAFT studies on massive stone and concrete. 

Thus, FT-SAFT reconstruction can image internal reflectors and block backwalls with high depth accuracy, enabling better structural analysis of the KC by combining calibrated shear wave velocities with the chosen bandwidth and filtering parameters. The results obtained provide reliable information on the block’s thickness, backwall echoes, and joint reflections of the examined block rows. The reflectors identified in this way allowed a systematic derivation of block depths and joint locations, which, in turn, formed the basis for the 3D model presented in this study..

Whereas previous work, such as^1,2^, and^3^, largely focused on the architectural geometry or construction of the Pyramid and the KC, the present study provides a more detailed insight into the internal structure of individual granite blocks of the KC. Although anomalies behind blocks such as NW9 have not yet been fully interpreted due to limited accessibility, the volumetric information provides a new basis for further numerical analyses, for example, of stress distribution, crack propagation, or the evaluation of hypotheses regarding additional voids or corridors. Data, computations, and evaluations of this quality may be used in the future to identify potential weaknesses in the masonry, thereby preventing damage to the Pyramid and avoiding hazards to visitors. Overall, the results show that the combination of the 8-channel and 16-channel UST devices with FT-SAFT reconstruction represents a powerful tool for non-destructive ultrasound testing of massive historic stone structures, contributing also to the preservation of the monument. Beyond this specific case study, the documented measurement strategy, processing workflow, and quantitative comparison between the two array configurations provide a methodological framework that can be applied to future NDT campaigns on heritage masonry structures.

## Conclusion and outlook

This study demonstrated that staged ultrasonic testing using complementary 8-channel and 16-channel UST devices enables reliable characterization of granite block thicknesses, backwalls, and internal reflectors within the KC. The derived reflector geometries enabled the construction of a volumetric 3D model from systematically reconstructed 2D profiles, providing a structural dataset at the block scale. 

Despite challenging environmental conditions, the combination of calibrated shear wave velocities and optimized reconstruction parameters yielded depth accuracies of a few centimeters for the main backwall reflections. By increasing the number of A-scans per measurement position from 28 (8-channel configuration) to 120 (16-channel configuration), the effective aperture and data density were substantially enhanced, which led to improved SNR, sharper backwall echoes, and a clearer representation of inclined and hyperbolic reflectors. The results indicate that advanced ultrasonic imaging can contribute not only to scientific investigation but also to long-term conservation by supporting structural assessment and risk evaluation in heritage monuments.

Nonetheless, several limitations remain. The interpretation of deeper reflectors is hampered by decreasing signal amplitude, local heterogeneity of the Aswan granite, and the inherent limitations of the FT-SAFT method. Some reflectors, particularly those near block boundaries or joints, may involve mode conversions or anisotropic effects that are not fully captured by the current imaging algorithm. In practical terms, limitations arise from the block’s partial inaccessibility, device dimensions and handling, especially with the 16-channel UST device, and the logistical constraints of working inside a frequently visited cultural monument.

A more comprehensive understanding of the KC’s internal structure requires additional information on boundary conditions, material variability, and potential multiple reflection paths within the granite block rows. Future work should therefore extend the ultrasonic approach through multimodal NDT campaigns integrating ground-penetrating radar (GPR), electrical resistivity tomography (ERT), infrared thermography, and high-resolution visual documentation. Image-fusion strategies^23^, successfully applied in previous SP investigations^7,14,24^ and in civil engineering applications^25^, may further enhance interpretability.

Advanced numerical modeling—including finite element analyses and elastic wave-propagation simulations^26^—should be employed to compare measured datasets with synthetic responses. Such comparisons may clarify whether observed reflectors are attributable to internal geometries, voids, stress redistribution, or microcracking.

Methodological refinements are also anticipated. Improvements in preprocessing, adaptive velocity modeling, and advanced reconstruction techniques, such as 3D-FT-SAFT^13,20^, full-waveform (FWI) inversion^27^ [], and full-matrix capture (FMC)-based acquisition systems^18^, are expected to further enhance resolution and structural insight. Beyond the specific case of the King’s Chamber, the documented screening-and-detail strategy, the velocity-calibrated FT-SAFT workflow, and the quantitative comparison of array configurations provide a transferable framework for ultrasonic investigations of other massive masonry or stone heritage structures. The continued integration of high-resolution ultrasonic datasets into refined 3D structural models will support future research, conservation planning, and exhibition-oriented visualization of the monument, and the staged 8-channel/16-channel strategy documented here can serve as a guideline for similar NDT investigations on other massive stone or masonry structures.

## Supplementary Information

Supplementary Information 1.

## Data Availability

Data will be made available upon request, subject to permission from the Egyptian Ministry of Tourism and Antiquities.

## Appendix

This appendix contains visualizations and information detailing the reconstruction process of the 3D model of the King’s Chamber, as shown in Fig. 7. In Fig. 8, all measurement profiles are shown and Figs. 9 through 15 illustrate the intermediate stages and outcomes of the data processing, showcasing how the 3D model was derived from visible reflections in reconstructed images.

### Overview of block depths

**Table 3:** Depth of reflections aligned with back-wall echoes of blocks of KC.

Block row	NW1	NW2	NW3	NW4	NW5	NW6	NW7	
Depth [m]	1.27	1.85	1.90	0.75	1.25	1.79	1.05	
Block row	NW8	NW9	NW10	NW11	NW12	Monolith		
Depth [m]	1.55	0.70	2.72	2.72	1.70	1.34		
Block row	EW1	EW2	EW3	EW4	EW5			
Depth [m]	-	1.05	2.00	1.40	1,25			
Block row	EW6	EW7	EW8					
Depth [m]	2.30	1.45	1.95					
Block row	SW3	SW4	SW5	SW6	SW7	SW8	SW9	SW10
Depth [m]	-	1.50	2.15	-	0.80	0.85	1.80	0.80
Block row	SW13	SW14	SW15	SW16	SW17	SW18	SW19	
Depth [m]	1.45	1.50	1.45	1.05	2.30	1.40	1.80	
Block row	WW1	WW2	WW3	WW4	WW5			
Depth [m]	1.94	1.25	2.30	1.50	1.25			
Block row	WW6	WW7	WW8	WW9				
Depth [m]	1.55	1.47	2.10	1.50				
