# Supplementary figures and images for: Investigation of the lower block rows in the King’s Chamber of the Great Pyramid using ultrasonic testing with shear wave arrays
Source: Sci Rep. 2026 Jun 18;16:19035. doi: 10.1038/s41598-026-54151-6 (PMC13279776; doi:10.1038/s41598-026-54151-6)

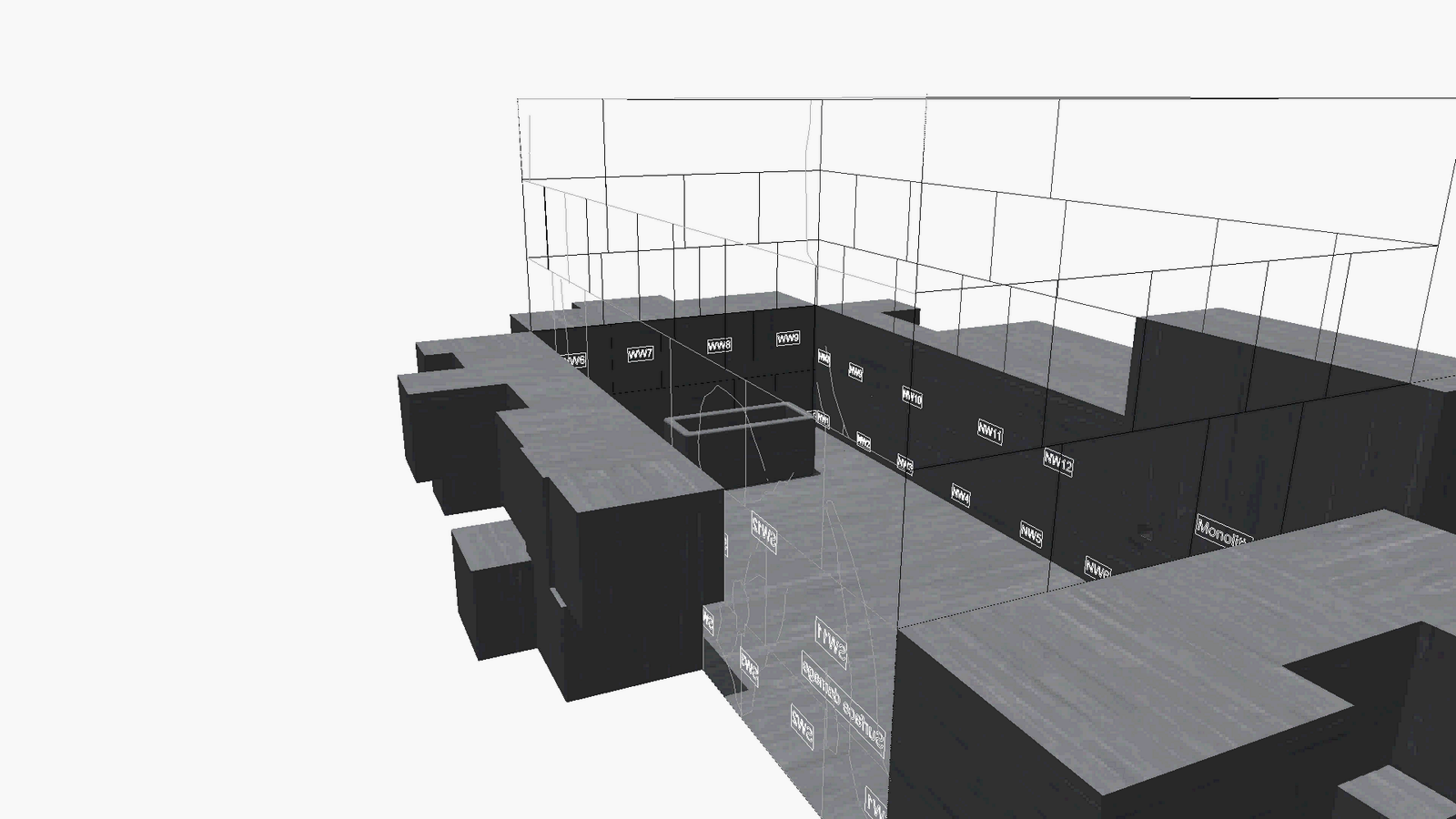

Supplement: Supplementary file 1 — Supplementary Information 1. [file 41598_2026_54151_MOESM1_ESM.gif]
